# Supplementary material for: Stepwise evolution of Salmonella Typhimurium ST313 causing bloodstream infection in Africa
Source: Nat Microbiol. 2020 Dec 21;6(3):327–38. doi: 10.1038/s41564-020-00836-1 (PMC8018540; doi:10.1038/s41564-020-00836-1)
Supplement: Supplementary file 1 — Supplementary Table 4, Supplementary Discussions 1 and 2, and Supplementary Methods. [file 41564_2020_836_MOESM1_ESM.pdf]

natureresearch

https://doi.org/10.1038/s41564-020-00836-1

Supplementary information

Stepwise evolution of *Salmonella* Typhimurium ST313 causing bloodstream infection in Africa

In the format provided by the authors and unedited

Supplementary Information for the manuscript "Stepwise evolution of *Salmonella* Typhimurium ST313 causing bloodstream infection in Africa."

Supplementary Table 1: Quality control statistics

Please see additional file

Supplementary Table 2: Metadata and accession numbers

Please see additional file

<u>Supplementary Table 3: Contextual metadata and accession numbers</u> [1–8]

Please see additional file

Supplementary Table 4: Table of pseudogenes including description, genotype and phenotype[2,3,9–16]

Gene Name	Gene Description	Genotypic Changes	Further evidence	ST19*	ST313 L1*	ST313 L3*	ST313 L2*
ratB **(STM2514)**	Secreted outer membrane protein associated with gut persistence in murine models.	ST313 L1: Q657*, Q1067H, T885I<br>ST313 L2: Q657*, Q1067H, R66C<br>ST313 L3: Q657*, Q1067H, R66C, G820S<br>Abundance of SNPs occur sporadically throughout the phylogeny.	Although a causal relationship is unproven, inactivation of *ratB* is suggested to have reduced the enteric potential of ST313, resulting in more systemic infections[9].				
katE **(STM1318)**	Stationary phase catalase involved in protecting high density bacterial communities in the environment from oxidative stress.	ST313 L1: E117G<br>ST313 L2: E117G<br>ST313 L3: E117G<br>Synonymous SNPs are additionally present in ST313 L1 (A→G at base 1452) and ST313 L3 (A→G at base 1908).	Reduction in catalase activity across all ST313[2,10]. We found that ST313 L3 had lower catalase activity than ST19 (this study).<br>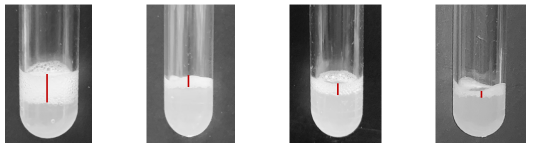<br>*bubbles represent catalase activity				
ttdA **(STM3355)**	L(+)-tartrate dehydratase involved in tartaric acid utilisation.	ST313 L1: Q68*<br>ST313 L2: Q68*<br>ST313 L3: Q68*<br>Additional mutations sporadically present across the phylogeny.	L-tartaric acid and dihydroxyacetone cannot be used as sole carbon sources by ST313. Results were originally generated with Biolog phenotype microarrays[9]. We found that ST313 L3 was also unable to grow on tartaric acid as a sole carbon source (this study).<br>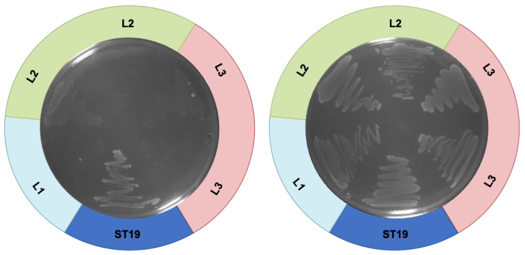				
melR **(STM4297)**	Melibiose operon response regulator involved in melibiose utilisation.	ST313 L2: F311L<br>ST313 L3: F311L<br>An additional synonymous SNP was also identified at base position 462 in ST313 L3.	ST313 L2 cannot grow on melibiose as a sole carbon source[11]. Assessment of the alpha-galactosidase activity of ST19 and ST313 wild-types and mutant strains showed that *melR* controlled the melibiose utilisation system[12]. We found that ST313 L3 was unable to grow on melibiose as a sole carbon source (this study).				

			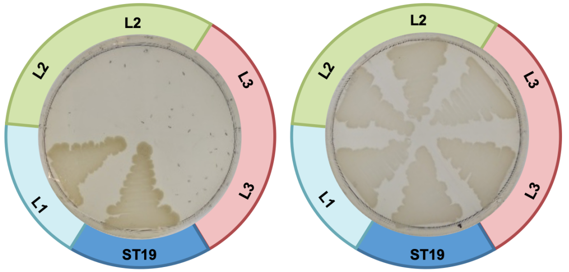<br><br>**Minimal Media + melibiose** **Minimal Media + glucose**				
flhA **(STM1913)**	Transmembrane biosynthesis protein involved in flagella protein export.	ST313 L2:A166T<br>ST313 L3:A166T	The A166T SNP caused reduced motility in ST313 L2, demonstrated by comparing D23580 *flhA*[474] mutant to wild type[12].				
pipD **(STM1094)**	Pathogenicity island-encoded protein D.	ST313 L2: 283bp deletion<br>ST313 L3: 283 bp deletion plus additional 109 bp deletion.	Although a causal relationship is unproven, the *pipD* gene contributes to macrophage persistence in murine models[9,13]. Of further interest, the *Salmonella* pathogenicity island SPI-5 gene *sopB* encodes a SPI-1 effector and has an associated chaperone *pipC.* In ST19 strains, these genes are upregulated in the gastrointestinal tract, which facilitates invasion into epithelial cells. However, in ST313 strains *sopB* and *pipC* are also upregulated in SPI-2 media and macrophages[12]. It is possible that this regulatory change is due to the loss of *orfX,* STM1093 and the 3' end of *pipD.*				
bcsG **(STM3624)**	Cellulose biosynthetic enzyme involved in biofilm formation.	ST313 L2: W247*	The RDAR phenotype of ST313 L2 is negative due to a mutation in *bcsG*[10]. The RDAR phenotype of ST313 L3 has an intermediate RDAR phenotype, however the genetic basis is unknown (this study).<br>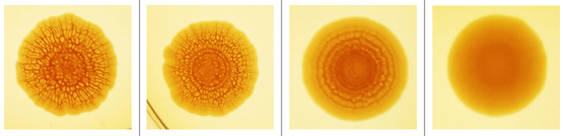<br>**ST19** **ST313 L1** **ST313 L3** **ST313 L2**<br>*RDAR colony morphology at 25°C				

sseI **(STM1051)**	Type III secretion system effector protein (SPI-2) involved in host cell dissemination.	In ST313 L2, the *sseI* gene contains an IS*26* transposase insertion. Generally, ST19, ST313 L1 and ST313 L3 strains carry a functional *sseI* gene. However, trends are not well conserved across lineages. For example, there are several ST313 L1 genomes which contain the IS*26* transposase.<br><br>Mutations in *sseI* are abundant and sporadic throughout all ST313 lineages. Specific non-synonymous mutations commonly occur in at least 10 amino acid positions; V13N, T47A, V69I, V70N, D81A, N86S, L91Q, D114Y, V115A and R266G.	Studies in mice have demonstrated that an accumulation of SNPs within *sseI* in ST313 L2 leads to an increased ability of ST313 L2 bacteria to disseminate rapidly from the gut to the draining lymph nodes in a murine model[14].				
lpxO **(STM4286)**	Putative membrane-bound beta-hydroxylase.	ST313 L2: E198*<br>An additional, synonymous SNP was also present across all ST313 lineages (G→T at base 411).	In D23580, *lpxO* is a pseudogene due to the presence of one SNP that introduces an early stop codon[3]. By mass spectrometry, we confirmed that the lipid A of D23580 was not modified by LpxO during growth in InSPI2 medium, while the lipid A of 4/74 contained this modification when grown in this medium. To confirm that LpxO-mediated modification of lipid A, we established that the mass spectrum of the lipid A from a 4/74 Δ*lpxO*::*aph* (KmR) mutant grown in InSPI2 was similar to the spectrum of D23580 grown in the same medium.<br><br>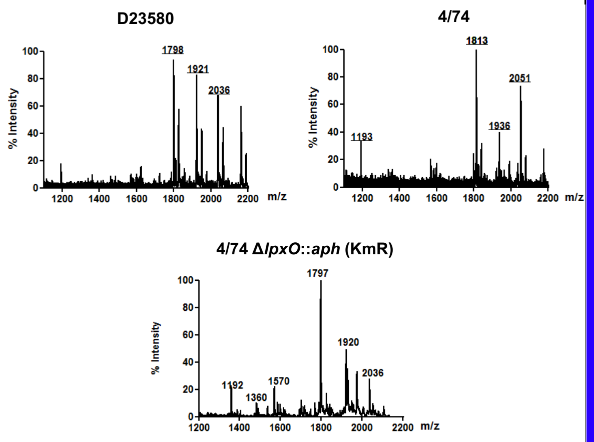 				

***macB* (STM0942)**	Putative ABC superfamily transport protein required to resist peroxide mediated killing	ST313 L1: W262*<br>ST313 L2 sub-lineage: Deletion of A and C at nucleotide position 444 and 445.	MacAB is involved in oxidative stress resistance[15] and mediates resistance to macrolide antibiotics[17]. Recently, ST313 L2-associated variants of the MacAB-TolC tripartite efflux pump have been shown to affect replication in macrophages, and influence fitness during colonisation of the murine gastrointestinal tract[16].				

* Note that predicted functionality (see methods) is depicted as a colour strip for each gene and is based on whole genome-based predictions of SNPs likely to play a functional role. Specifically, blue indicates functional and pink indicates non-functional.

Supplementary Table 5: Mutations in top predictor genes for invasiveness

Please see additional file

Supplementary Discussion 1: pAnkS characterisation[3,18,19]

Of interest we identified an 8,274 bp novel plasmid in ST313 L1 which shares 99.27% sequence identity with plasmid pAnkS[31] (Supplementary Discussion 1 Figure 1). The corresponding plasmid in ST313 L1 (pAnkS$^{ST313\text{-}L1}$) carries a functional *lsoAB* type 2 toxin-antitoxin (TA) system, while *lsoA* (encoding for the toxin LsoA) in the originally described pAnkS (GenBank: NC_010896.1) is interrupted by a premature stop codon (**C**AG^{Q} →**T**AG^{STOP}, position 1741 in pAnkS). The LsoAB of pAnkS$^{ST313\text{-}L1}$ have 100% sequence identity to the LsoAB proteins found in pOSAK1, a cryptic plasmid of enterohaemorrhagic *Escherichia coli* O157:H7[18].

To understand the role of the novel plasmid repertoire associated with ST313 L1, we performed functional experiments. In *E. coli*, the LsoA endoribonuclease acts as a toxin involved in resistance to the Dmd negative mutant of the *E. coli* bacteriophage (phage) T4 (*Myoviridae* family)[18,19]. This observation suggests a broader role of the LsoAB TA system in phage immunity and we investigated the role of pAnkS$^{ST313\text{-}L1}$ in *Salmonella* phage exclusion. We transferred pAnkS$^{ST313\text{-}L1}$ from *S.* Typhimurium ST313 L1 strain A130[3] into *S.* Typhimurium strain LT2 (ST19), a strain commonly used in phage biology. We used a double-layer plaque assay to assess the phage susceptibility of strain LT2, in comparison with LT2 carrying pAnkS$^{ST313\text{-}L1}$. We assessed the phage susceptibility of LT2 with and without pAnkS$^{ST313\text{-}L1}$ using a range of temperate and virulent phages. We discovered that pAnkS$^{ST313\text{-}L1}$ conferred resistance to the virulent phages Det7 (*Akermannviridae* family) and S16 (*Myoviridae* family). Specifically, the phage efficiency of plating and plaque size were drastically reduced by the presence of pAnkS$^{ST313\text{-}L1}$ (Supplementary Discussion 1 Figure 1). To confirm the involvement of LsoAB in the phage exclusion phenotype, we excised the *lsoAB* from pAnkS$^{ST313\text{-}L1}$, generating the plasmid pAnkS$^{ST313\text{-}L1}$Δ*lsoAB*. As expected, pAnkS$^{ST313\text{-}L1}$Δ*lsoAB* did not confer resistance to phages Det7 and S16 in LT2 (Supplementary Discussion 1, Figure 1). Taken together, these results demonstrate a protective role of pAnkS$^{ST313\text{-}L1}$ against phage predation in *S.* Typhimurium ST313 L1. This ability to resist phage killing could enhance survival of ST313 L1 in certain environmental niches.

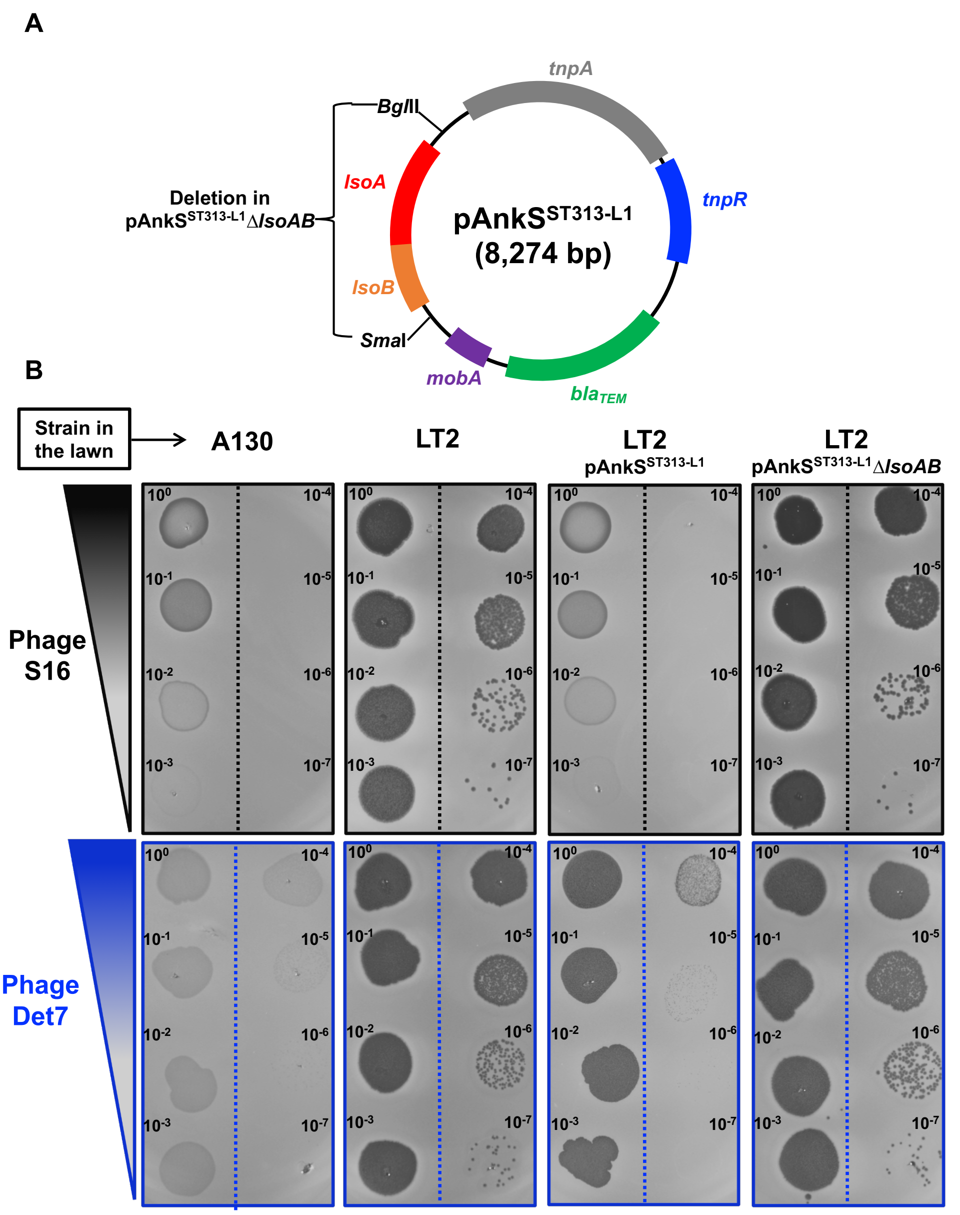

Supplementary Discussion 1 Figure 1: The LsoAB toxin-antitoxin system of pAnkS$^{ST313\text{-}L1}$ confers phage immunity in *Salmonella*. (**A**) Schematic of pAnkS$^{ST313\text{-}L1}$ of strain S. Typhimurium strain A130. Gene annotation is based on the previously described plasmids pAnkS (GenBank: NC_010896.1) and pOSAK1 (GenBank: AB011548.2). The restriction sites *Bgl*II and *Sma*I, used to remove *lsoAB* in plasmid pAnkS$^{ST313\text{-}L1}$Δ*lsoAB* are indicated. (**B**) *lsoAB* confers resistance to virulent phages S16 and Det7. Plaque assays were carried out with 10 µl of decimal dilutions of phage stocks (10^{10} CFU/ml) applied on lawns of *Salmonella* strains A130, LT2, LT2/pAnkS$^{ST313\text{-}L1}$ and LT2/pAnkS$^{ST313\text{-}L1}$Δ*lsoAB*. This screening revealed that pAnkS$^{ST313\text{-}L1}$ did not confer resistance to the temperate phages P22, BTP1, ES18, or the virulent phages FelixO1, 9NA, Chi, or SP6 (data not shown). Phage dilution factors are indicated.

Supplementary Discussion 2: AMR Discussion[6,20,21]

To determine AMR trends, we examined the 680 *S.* Typhimurium isolates for genomic determinants of resistance. To confirm genome-based predictions experimentally, we calculated the sensitivity and specificity of our genome predictions phenotypically using 528 available isolates (measured by EUCAST breakpoint). For chloramphenicol; sensitivity was 96.71 % (95 % Confidence Interval [CI] = 94.53 % to 98.19 %) and specificity was 83.60 % (95 % CI = 78.42 % to 87.97 %). For ampicillin; sensitivity was 98.04 % (95 % CI = 96.52 % to 99.02 %) and specificity was 95.61 % (95 % CI = 90.06 % to 98.56 %). For cotrimoxazole; sensitivity was 97.38 % (95 % CI = 95.56 % to 98.60 %) and specificity was 77.09 % (95 % CI = 70.24 % to 83.03 %). Taken together, our data reiterates previous findings in *S.* Typhimurium that show genome-based analysis accurately predicted the AMR phenotypes of 89.8 % isolates, with 83% sensitivity and 96% specificity[22].

Sub-sampling of a comprehensive archive allowed us to capture the full range of AMR profiles in *S.* Typhimurium responsible for BSI in Malawi between 1996 and 2018. Across the sampling period there were changes in antimicrobial usage policies at the local level (Queen Elizabeth Central Hospital) in Malawi, including the phased removal of chloramphenicol from clinical practice. From approximately 2002 onwards, chloramphenicol was replaced by the oral fluoroquinolone, ciprofloxacin for treatment of culture-confirmed iNTS disease. Additionally, from 2005, the Department of Medicine Malawi began to use the 3rd-generation cephalosporin, ceftriaxone for the empirical management of suspected sepsis[20]. More recently, chloramphenicol has largely been removed from hospital and private pharmacies suggesting that it is infrequently used to treat any infection (M. Gordon, personal communication). Across our sampling period there has been a reduction in the proportion of chloramphenicol-resistant isolates of ST313 L2, raising the possibility that a change in policy on empirical chloramphenicol use has played a role in shaping the epidemiology of ST313 *S*. Typhimurium in Malawi.

Combining the available details on antimicrobial usage with fluctuating AMR profiles allows us to speculate on trends in circulating *Salmonella* lineages, particularly the switch from MDR ST313 L2 to the pan-susceptible ST313 L3. We hypothesise that the phased removal of chloramphenicol from clinical practice following local policy changes has opened a window of opportunity for the emergence of fully susceptible ST313 L3 as a clinical problem in Malawi. A comprehensive epidemiological study on antimicrobial usage would be required to properly investigate this hypothesis.

Recently, an XDR ST313 L2 sub-lineage was identified in the Democratic republic of Congo[6]. We did not identify any isolates displaying an XDR genotype in our collection. However, we did identify five isolates that have a single *gyrA* mutation associated with reduced susceptibility to ciprofloxacin, which were ST313 L2 strains isolated in Malawi (*n*=3), Mali (*n*=1) or Cameroon (*n*=1). The oldest fluoroquinolone-resistant isolate originated from Cameroon in 1998, carried a single *gyrA* mutation (S83F) and was sequence-typed as ST19 despite belonging to ST313 L2. The Mali isolate, dated from 2008, carried the same *gyrA* mutation. The three Malawi isolates carried either the *gyrA* (D87N) or *gyrA* (D87Y) mutations, and were isolated after ciprofloxacin was introduced to treat iNTS disease (specifically in 2008, 2013 and 2018). In 2016, the Queen Elizabeth Central Hospital in Blantyre (Malawi) successfully introduced an antimicrobial stewardship programme, which has been effective in improving usage of third generation cephalosporins[21]. Ongoing prospective surveillance will now be important to flag any rises in ciprofloxacin and ceftriaxone resistance in ST313 lineages.

Supplementary Methods: Microbiological materials and methods[2,3,10,23–33]

Catalase assay

To compare catalase activity of ST313 lineage-representatives, a catalase assay was performed based on methods outlined previously[2,10]. Briefly, 10 µL of 6% aqueous H_2O_2 was added to 1 cm diameter glass test tubes containing 1ml of bacterial overnight cultures grown in LB (Lennox). Tubes were photographed after 5 minutes incubation at room temperature and bubble height was compared.

Melibiose and tartrate utilisation

To determine melibiose and tartrate utilisation as sole carbon sources, a *Salmonella* culture was grown overnight in LB (Lennox) broth at 37°C. A 1 mL aliquot was centrifuged at 13,000 x*g* for 5 min and the supernatant discarded. The cell pellet was washed twice with 1 mL of PBS and streaked on minimal M9 medium (1×M9 Minimal Salts (Sigma Aldrich), 2 mM MgSO4, and 0.1 mM CaCl2) plates supplemented with 0.4 % of either melibiose or tartrate, and minimal M9 medium plates supplemented with 0.2 % glucose as control. Plates supplemented with melibiose (and glucose control) were incubated overnight at 37°C. Plates supplemented with tartrate (and glucose control) were also supplemented with 20 mM Trimethylamine N-oxide dihydrate (TMAO) as an electron acceptor, and incubated for 5 days at 30°C under anaerobic conditions using gas packs (AnaeroGen 2.5 L sachets, Thermo Scientific) and a resazurin indication (Thermo Scientific, Oxoid).

RDAR morphotype

Red Dry and Rough (RDAR) phenotype was assessed to determine production of extracellular matrix components curli and cellulose[10,28]. A *Salmonella* culture was grown overnight in LB (Lennox) broth at 37°C, and an aliquot of 10 µL was dropped onto LB agar without NaCl, supplemented with 40 µg/mL Congo red and 20 µg/mL Coomassie Brilliant Blue. Plates were incubated at room temperature for 7 days.

Construction of the *S*. Typhimurium 4/74 Δ*lpxO*::*aph* mutant by λ Red recombineering

The Lambda Red recombineering technology[29] was used to delete the *lpxO* gene from the *S*. Typhimurium 4/74 chromosome. Primers Fw-lpxO-P1 (ATGTTCGCAGCAATCATTATCGGTATTTTTATTATCAGCGTCATTGTGTAGGCTGGAGCTGCTTC) and Rv-lpxO-P2 (AATAGCAGCAATAACGAGATATTTCAGCGCGTAATAGAGAGTGCGCATATGAATATCCTCCTTAG) were used for amplification of the kanamycin resistance cassette (*aph*) from pKD4. The resulting PCR fragment was electroporated into 4/74 carrying the recombineering plasmid pSIM5-*tet* following methods previously described[30]. When 4/74 pSIM5-*tet* bacterial culture reached mid-exponential phase (OD_{600nm} 0.3–0.4 at 30°C) in LB supplemented with tetracycline, heat treatment (42°C, 15 min) was used to induce the λ Red operon. Recombinant candidates were selected on LB-agar kanamycin 50 µg/mL. The Δ*lpxO*::*aph* mutation was transduced into wild-type 4/74 using the high-frequency-transducing bacteriophage P22 HT 105/1 *int-201*[31].

Bacterial growth and sample preparation for lipid A analysis by mass spectrometry

Bacteria were grown in LB (Lennox) broth or SPI-2-inducing (InSPI2) growth condition at 37°C 220 rpm in a water bath. For growth in Lennox broth, two growth conditions were tested: early stationary phase (ESP), growth to OD_{600nm} 2; and late stationary phase (LSP), growth to OD_{600nm} 2 followed by a further 6 h growth in the same incubation conditions. Lennox broth consisted in 10 g/L tryptone, 5 g/L yeast extract, and 5 g/L NaCl. PCN medium. The InSPI2 growth condition involved growth in phosphate carbon nitrogen (PCN) minimal medium[32] at pH 5.8 and 0.4 mM Pi to OD_{600nm} 0.3[33]. Pellets from bacterial cultures were washed twice with phosphate buffer (10.14 mM Na_2HPO_4, 1.76 mM KH_2PO_4, adjusted to pH 7.4) and lyophilized.

Mass Spectrometry Analysis

Lipid A molecules were extracted using an ammonium hydroxide-isobutyric acid method[24] and subjected to negative-ion matrix-assisted laser desorption ionization (MALDI)–time of flight (TOF) mass spectrometry analysis. 1-2 mgr of lyophilized bacteria were resuspended in 400 µl of isobutyric acid–1 M ammonium hydroxide (5:3 [vol/vol]) in a screw-cap test tube, and incubated at 100°C for 2 h before being cooled in ice water and centrifuged (2,000 × *g* for 15 min). The supernatant was transferred to a new tube, diluted with an equal volume of water, and lyophilized. The lyophilized material was washed twice with 400 µl of methanol and centrifuged (2,000 × *g* for 15 min). The insoluble lipid A was solubilized in 50 to 100 µl of chloroform-methanol-water (3:1.5:0.25 [vol/vol/vol]). To analyze the samples, a few microliters of the lipid A suspension (1 mg/ml) was desalted with a few grains of ion-exchange resin (H^+; Dowex 50W-X8) in a 1.5-ml microcentrifuge tube. A 1-µl aliquot of the suspension (50 to 100 µl) was deposited on the target and covered with the same amount of dihydroxybenzoic acid matrix (Bruker Daltonics Inc.) dissolved in acetonitrile–0.1% trifluoroacetic acid (1:2 [vol/vol]). Different ratios between the samples and dihydroxybenzoic acid were used when necessary. Analyses were performed on a Bruker Autoflex speed TOF/TOF mass spectrometer (Bruker Daltonics Inc.) in negative reflective mode with delayed extraction. The ion-accelerating voltage was set at 20 kV. Each spectrum was an average of 300 shots. A peptide calibration standard (Bruker Daltonics Inc.) was used to calibrate the MALDI-TOF/TOF mass spectrometer. Further calibration for lipid A analysis was performed externally using lipid A extracted from *E. coli* strain MG1655 grown in LB medium at 37°C. Spectra are representative of three independent lipid A extractions.

Polymerase Chain Reaction (PCR) of selected plasmids in *Salmonella* Typhimurium isolates

To confirm genotypic predictions, colony PCR of *Salmonella* plasmids was performed. PCR reactions were carried out on the GENEFLOW SENSOQUEST labcycler with MyTaq Red Mix 1X (Bioline, UK, BIO-25043), according to manufacturer's recommendations. DNA oligonucleotides (primers) were synthesised by EuroFins Genomics (table below). PCR cycle conditions were as follows: 95°C for 2

minutes, x35(95°C for 15 seconds, 55°C for 30 seconds, 72°C for 1 minute 30 seconds), 72°C for 5 minutes. DNA from PCR products were analysed by DNA electrophoresis on 1% molecular grade agarose gels (Bioline, BIO-41025) in TAE 1X buffer supplemented with 4µl/100 ml of Midori Green (Nippon Genetics, Germany MG 04). To estimate the PCR product size, 1Kb ladders (Bioline, H1-819101A) were used. Electrophoresis was run at 100V for 60min and DNA bands were visualised using the SYNGENE Bio imaging system.

Supplementary Methods Table 1: Primers used in this study

Plasmid	Direction	Sequence
pSLT/pSLT-BT	Forward	ACATAATGCAGAATAAAAGATCGCT
	Reverse	AGTGTCCTGCCCAAAGCCTCTG
Tn21 flanking region in pSLT-BT	Forward	CACCCAGCATATCGAGGAAG
	Reverse	TACACCCAAAACGCACAGTC
pBT1	Forward	GGATTCGTACACCCGCCTC
	Reverse	TCGGTGGCCGTTCGTCTCG
pBT2	Forward	GGCTGAATGGTGTGAATTTCC
	Reverse	GAAAGCTACTGCGATTGTTTCC
pBT3	Forward	AACCATCACCTGTTAGCAGTG
	Reverse	ATGACTGACACGAATTCTACC
pAnkS	Forward	AGACGCTCTTCCGATAGTGG
	Reverse	AACCCGGTAAGACACGACTT

Isolation and modification of plasmid pAnkS$^{ST313-L1}$

All the enzymatic reactions were carried out with reagents purchased from New England Biolabs (NEB, MA, USA), according to the manufacturer recommendations. DNA and plasmid purification kits were purchased from Bioline (BIO-5205, BIO-52060).

The ampicillin resistant plasmid pAnkS$^{ST313-L1}$ (8,274 bp) was extracted from *S.* Typhimurium ST313 L1 strain A130[3] and chemically competent *E. coli* Top10 (Invitrogen) were transformed with the resulting plasmid prep[25]. The pAnkS$^{ST313-L1}$ transformants were selected with ampicillin (100 µg/ml). The plasmid was extracted from a Top10/ pAnkS$^{ST313-L1}$ culture and digested with restriction enzymes BglII and SmaI to excise the *lsoAB* fragment (1.55 Kb). The digestion was analysed by agarose gel electrophoresis and the 6.72 Kb DNA fragment was purified and treated with T4 DNA polymerase in the presence of dNTPs, to blunt-end the *Bgl*II cohesive-ends. After purification, the fragment was self-ligated, using T4 DNA

ligase and the ligation reaction was transformed into *E. coli* Top10. After selection on ampicillin, the resulting plasmid pAnkS$^{ST313\text{-}L1}$Δ*lsoAB* (6,719 bp) was extracted and verified by restriction analysis with BamHI. Plasmids pAnkS$^{ST313\text{-}L1}$and pAnkS$^{ST313\text{-}L1}$Δ*lsoAB* were transferred by electroporation[26] into *S.* Typhimurium strain LT2[27], resulting in the ampicillin resistant strains LT2/pAnkS$^{ST313\text{-}L1}$ and LT2/pAnkS$^{ST313\text{-}L1}$Δ*lsoAB*.

Bacteriophage manipulation and double-layer plaque assay

All the bacteriophage (phage) stocks were prepared in LB (Lennox) with the prophage-cured strain *S.* Typhimurium D23580ΔΦ (JH3949) as host[23]. Ten millilitre cultures of exponentially growing JH3949 were infected with ~10^5 Plaque Forming Units (PFU) of each phage and incubated at 37°C with shaking (220 rpm) for at least 3 hours. After centrifugation (4,000 X *g*, 15 min) the supernatants were filtered (0.22 µm, StarLab). The resulting phage lysates (10 ml) were mixed with 100 µl of chloroform to prevent bacterial contamination and were stored at 4°C. Each lysate was serial-diluted and phage enumeration was performed by double-layer plaque assay, as previously described[23], with LB Top Agar (0.5% agar in LB) and the reporter strain JH3949. Stock lysates of phages P22, BTP1, ES18, 9NA, FelixO1, Chi, Sp6, S16 and Det7 were adjusted to ~10^{10} PFU/ml and decimal dilutions were prepared to 10^{-7}. To assess phage susceptibility by plaques assay, 10 µl of each phage dilution was applied on bacterial Lawns of strains A130, LT2, LT2/pAnkS$^{ST313\text{-}L1}$ and LT2/pAnkS$^{ST313\text{-}L1}$Δ*lsoAB*. Pictures of the resulting plaques were taken with an ImageQuant Las 4000 imager (GE Healthcare) after 16-20 hours of incubation at 37°C.

References for Supplementary Information

1. Almeida, F. *et al.* Multilocus sequence typing of Salmonella Typhimurium reveals the presence of the highly invasive ST313 in Brazil. *Infect. Genet. Evol.* **51**, 41–44 (2017).
2. Ashton, P. M. *et al.* Public health surveillance in the UK revolutionises our understanding of the invasive Salmonella Typhimurium epidemic in Africa. *Genome Med.* **9**, 92 (2017).
3. Kingsley, R. A. *et al.* Epidemic multiple drug resistant Salmonella Typhimurium causing invasive disease in sub-Saharan Africa have a distinct genotype. *Genome Res.* **19**, 2279–2287 (2009).
4. Msefula, C. L. *et al.* Genotypic Homogeneity of Multidrug Resistant S. Typhimurium Infecting Distinct Adult and Childhood Susceptibility Groups in Blantyre, Malawi. *PLoS One* **7**, e42085 (2012).
5. Okoro, C. K. *et al.* Intracontinental spread of human invasive Salmonella Typhimurium pathovariants in sub-Saharan Africa. *Nat. Genet.* **44**, 1215–1221 (2012).
6. Van Puyvelde, S. *et al.* An African Salmonella Typhimurium ST313 sublineage with extensive drug-resistance and signatures of host adaptation. *Nat. Commun.* **10**, 4280 (2019).
7. Jacob, J. J. *et al.* Genomic analysis of human invasive Salmonella enterica serovar Typhimurium ST313 isolate B3589 from India. *Infect. Genet. Evol.* **73**, 416–424 (2019).
8. Tran-Dien, A., Le Hello, S., Bouchier, C. & Weill, F.-X. Early transmissible ampicillin resistance in zoonotic Salmonella enterica serotype Typhimurium in the late 1950s: a retrospective, whole-genome sequencing study. *Lancet Infect. Dis.* **18**, 207–214 (2018).
9. Okoro, C. K. *et al.* Signatures of adaptation in human invasive Salmonella Typhimurium ST313 populations from sub-Saharan Africa. *PLoS Negl. Trop. Dis.* **9**, e0003611 (2015).
10. Singletary, L. A. *et al.* Loss of Multicellular Behavior in Epidemic African Nontyphoidal Salmonella enterica Serovar Typhimurium ST313 Strain D23580. *MBio* **7**, e02265 (2016).
11. Yang, J. *et al.* Characterization of the Invasive, Multidrug Resistant Non-typhoidal Salmonella Strain D23580 in a Murine Model of Infection. *PLoS Negl. Trop. Dis.* **9**, e0003839 (2015).
12. Canals, R. *et al.* Adding function to the genome of African Salmonella Typhimurium ST313 strain D23580. *PLOS Biol.* **17**, e3000059 (2019).
13. Lawley, T. D. *et al.* Genome-Wide Screen for Salmonella Genes Required for Long-Term Systemic Infection of the Mouse. *PLOS Pathog.* **2**, e11 (2006).
14. Carden, S. E. *et al.* Pseudogenization of the Secreted Effector Gene sseI Confers Rapid Systemic Dissemination of S. Typhimurium ST313 within Migratory Dendritic Cells. *Cell Host Microbe* **21**, 182–194 (2017).
15. Bogomolnaya, L. M. *et al.* The ABC-Type Efflux Pump MacAB Protects Salmonella enterica serovar Typhimurium from Oxidative Stress. *MBio* **4**, e00630-13 (2013).
16. Honeycutt, J. D. *et al.* Genetic variation in the MacAB-TolC efflux pump influences pathogenesis of invasive Salmonella isolates from Africa. *PLOS Pathog.* **16**, e1008763 (2020).
17. Nishino, K., Latifi, T. & Groisman, E. A. Virulence and drug resistance roles of multidrug efflux systems of Salmonella enterica serovar Typhimurium. *Mol. Microbiol.* **59**, 126–141 (2006).
18. Otsuka, Y. & Yonesaki, T. Dmd of bacteriophage T4 functions as an antitoxin against Escherichia coli LsoA and RnlA toxins. *Mol. Microbiol.* **83**, 669–681 (2012).
19. Wan, H. *et al.* Structural insights into the inhibition mechanism of bacterial toxin LsoA by bacteriophage antitoxin Dmd. *Mol. Microbiol.* **101**, 757–769 (2016).
20. Feasey, N. A. *et al.* Three Epidemics of Invasive Multidrug-Resistant Salmonella Bloodstream Infection in Blantyre, Malawi, 1998-2014. *Clin. Infect. Dis.* **61 Suppl 4**, S363-71 (2015).
21. Lester, R. *et al.* Sustained reduction in third-generation cephalosporin usage in adult inpatients following introduction of an antimicrobial stewardship program in a large urban hospital in Malawi. *Clin. Infect. Dis.* (2020). doi:10.1093/cid/ciaa162
22. Mensah, N. *et al.* Determining antimicrobial susceptibility in Salmonella enterica serovar Typhimurium through whole genome sequencing: a comparison against multiple phenotypic susceptibility testing methods. *BMC Microbiol.* **19**, 148 (2019).
23. Owen, S. V *et al.* Characterization of the Prophage Repertoire of African Salmonella Typhimurium ST313 Reveals High Levels of Spontaneous Induction of Novel Phage BTP1. *Front. Microbiol.* **8**, 235 (2017).
24. Llobet, E. *et al.* Deciphering tissue-induced Klebsiella pneumoniae lipid A structure. *Proc. Natl. Acad. Sci. U. S. A.* **112**, E6369-78 (2015).
25. Green, R. & Rogers, E. J. Transformation of chemically competent E. coli. *Methods Enzymol.* **529**, 329–

336 (2013).
26. O'Callaghan, D. & Charbit, A. High efficiency transformation of Salmonella typhimurium and Salmonella typhi by electroporation. *Mol. Gen. Genet. MGG* **223**, 156–158 (1990).
27. McClelland, M. *et al.* Complete genome sequence of Salmonella enterica serovar Typhimurium LT2. *Nature* **413**, 852–856 (2001).
28. Ramachandran, G., Aheto, K., Shirtliff, M. E. & Tennant, S. M. Poor biofilm forming ability and long-term survival of invasive Salmonella Typhimurium ST313. *Pathog. Dis.* ftw049 (2016). doi:10.1093/femspd/ftw049
29. Datsenko, K. A. & Wanner, B. L. One-step inactivation of chromosomal genes in Escherichia coli K-12 using PCR products. *Proc. Natl. Acad. Sci.* **97**, 6640 LP – 6645 (2000).
30. Koskiniemi, S., Pranting, M., Gullberg, E., Nasvall, J. & Andersson, D. I. Activation of cryptic aminoglycoside resistance in Salmonella enterica. *Mol. Microbiol.* **80**, 1464–1478 (2011).
31. Schmieger, H. Phage P22-mutants with increased or decreased transduction abilities. *Mol. Gen. Genet. MGG* **119**, 75–88 (1972).
32. Lober, S., Jackel, D., Kaiser, N. & Hensel, M. Regulation of Salmonella pathogenicity island 2 genes by independent environmental signals. *Int. J. Med. Microbiol.* **296**, 435–447 (2006).
33. Kroger, C. *et al.* An infection-relevant transcriptomic compendium for Salmonella enterica Serovar Typhimurium. *Cell Host Microbe* **14**, 683–695 (2013).
