# Supplementary material for: Stepwise evolution of Salmonella Typhimurium ST313 causing bloodstream infection in Africa
Source: Nat Microbiol. 2020 Dec 21;6(3):327–38. doi: 10.1038/s41564-020-00836-1 (PMC8018540; doi:10.1038/s41564-020-00836-1)
Supplement: Supplementary file 2 — Reporting Summary [file 41564_2020_836_MOESM2_ESM.pdf]

nature research

Corresponding author(s): Jay C. D. Hinton
Last updated by author(s): Nov 12, 2020

Reporting Summary

Nature Research wishes to improve the reproducibility of the work that we publish. This form provides structure for consistency and transparency in reporting. For further information on Nature Research policies, see our Editorial Policies and the Editorial Policy Checklist.

Statistics

For all statistical analyses, confirm that the following items are present in the figure legend, table legend, main text, or Methods section.

n/a	Confirmed	
☐	☒	The exact sample size (*n*) for each experimental group/condition, given as a discrete number and unit of measurement
☒	☐	A statement on whether measurements were taken from distinct samples or whether the same sample was measured repeatedly
☐	☒	The statistical test(s) used AND whether they are one- or two-sided<br>*Only common tests should be described solely by name; describe more complex techniques in the Methods section.*
☐	☒	A description of all covariates tested
☒	☐	A description of any assumptions or corrections, such as tests of normality and adjustment for multiple comparisons
☐	☒	A full description of the statistical parameters including central tendency (e.g. means) or other basic estimates (e.g. regression coefficient) AND variation (e.g. standard deviation) or associated estimates of uncertainty (e.g. confidence intervals)
☐	☒	For null hypothesis testing, the test statistic (e.g. *F*, *t*, *r*) with confidence intervals, effect sizes, degrees of freedom and *P* value noted<br>*Give P values as exact values whenever suitable.*
☐	☒	For Bayesian analysis, information on the choice of priors and Markov chain Monte Carlo settings
☒	☐	For hierarchical and complex designs, identification of the appropriate level for tests and full reporting of outcomes
☒	☐	Estimates of effect sizes (e.g. Cohen's *d*, Pearson's *r*), indicating how they were calculated

Our web collection on statistics for biologists contains articles on many of the points above.

Software and code

Policy information about availability of computer code

Data collection	Software used for data collection; Microsoft Excel for Mac v 16.29.1
Data analysis	Software used for data analysis is as follows; Trimmomatic v0.36, Seqtk v1.2-r94, Fastqc v0.11.5, Multiqc v1.0, Unicycler v0.3.0 and v0.4.0, QUAST v4.6.3, Prokka v1.12, SISTR v1.0.2, MLST v2.10 , BWA mem v0.7.10-r789, SAMtools v1.7, GATK v3.7, Picard v2.10.1-SNAPSHOT, Bcftools v1.9-80, QualiMap v2.0, Gubbins v2.2, RAxML-ng v0.6.0, rhierBAPs v1.1.3, Interactive Tree Of Life v4.2, Roary v3.11.0, SNP-sites v2.3.3, BEAUTI v2.6.1, BEAST2 v2.6.1, Tracer v1.7.1, LogCombiner v2.6.1, DensiTree v2.2.7, TreeAnnotator v2.6.0 , R v3.4.0, SRST2 v0.2.0, MegaX v0.1, staramr v0.5.1, BLAST v2.6.0, Guppy v3.1.5, Artemis Comparison Tool v13.0.0, Invasiveness Index [available at https://github.com/Gardner-BinfLab/invasive_salmonella], Microsoft Excel for Mac v 16.29.1.

For manuscripts utilizing custom algorithms or software that are central to the research but not yet described in published literature, software must be made available to editors and reviewers. We strongly encourage code deposition in a community repository (e.g. GitHub). See the Nature Research guidelines for submitting code & software for further information.

Data

Policy information about availability of data

All manuscripts must include a data availability statement. This statement should provide the following information, where applicable:

- Accession codes, unique identifiers, or web links for publicly available datasets
- A list of figures that have associated raw data
- A description of any restrictions on data availability

Sequence data that support the findings of this study have been deposited in the Sequence Read Archive (https://www.ncbi.nlm.nih.gov/sra). Accession numbers are available in Table S2. The complete genome and plasmid sequence for ST313 L3 strain BKQZM9 can be found under bioproject ID PRJNA656707, specifically complete chromosome (GenBank accession: CP060169), pSLT (GenBank Accession: CP060170), pBT3 (GenBank Accession: CP060171). Publicly available sequence

data were downloaded from one of the following sources: GenBank (https://www.ncbi.nlm.nih.gov/genbank/), Sequence Read Archive (https://www.ncbi.nlm.nih.gov/sra), European Nucleotide Archive (https://www.ebi.ac.uk/ena) or Enterobase (https://enterobase.warwick.ac.uk). Accession numbers for contextual isolates are listed in Table S3. The data underlying Figures 1, 2, 3, 4, 5, S1 and S2 are available in Table S2. Additional data underlying Figure S2 are available in Table S3. The remaining relevant data are within the manuscript and its supporting information files.

Field-specific reporting

Please select the one below that is the best fit for your research. If you are not sure, read the appropriate sections before making your selection.

☒ Life sciences ☐ Behavioural & social sciences ☐ Ecological, evolutionary & environmental sciences

For a reference copy of the document with all sections, see nature.com/documents/nr-reporting-summary-flat.pdf

Life sciences study design

All studies must disclose on these points even when the disclosure is negative.

Sample size

Samples were derived from two archived blood culture collections to achieve the largest collection of African Salmonella Typhimurium bloodstream isolates sequenced to date.

The main dataset was sourced from from the Malawi Liverpool Wellcome Clinical Research Programme (MLW) in Blantyre, Malawi which consists of ~8,000 Salmonella Typhimurium bloodstream isolates from patients with iNTS disease. Due to budgetary and staffing limitations, the maximum number of samples which could be whole genome sequenced was 1,000.

The second collection comprised of a contextual dataset sourced from the Institut Pasteur, Paris and consists of 94 Salmonella Typhimurium isolates collected from human extraintestinal sites of patients. All 94 samples were sent for whole genome sequencing.

Data exclusions

Data was excluded from downstream analysis in the following cases;

Any bacterial sample which was unable to be resuscitated from freezer archives.

Any sample which was identified as a serovar other than Salmonella Typhimurium following computational typing of genome sequence data.

Any sample which failed quality control of sequence reads. Specifically if the sample failed basic quality statistics, per base sequence quality, per base N content, adapter content or had an average GC content out of acceptable range (47 % - 57 %).

Any assembly which failed quality control. Specifically, if the sample had an N50 > 20 kb, had 600 or more contiguous sequences or the total number of bases was outside of acceptable range (between 4Mbp and 5.8Mbp).

Any sequenced sample which failed mapping quality control. Specifically, if the mapped sample had a coverage depth less than 10x.

Replication

To verify study reproducibility, the results of all automated computational approaches were confirmed either at the phenotypic level and/or by using multiple bioinformatic strategies. In addition, all phenotypic testing was performed in triplicate and each of the phenotypes was fully reproducible. To generate robust maximum likelihood phylogenetic trees, bootstrapping was used to assess branch support. Bayesian evolutionary analysis (BEAST) was run on three independent chains, each of length 250,000,000. All attempts at replication were successful.

Randomization

For the MLW collection, isolates listed as S. Typhimurium were extracted from the metadata file and stratified by antimicrobial resistance profile into the following four categories: susceptible, resistant to one first line agent, resistant to two first line agents or multi-drug resistant. Sub-sampling was then performed on each strata using a random number generator (Excel, Microsoft) to select 1,000 isolates. Randomization was not necessary for the contextual collection, as all samples were included in the study.

Blinding

Data collection was performed by a diagnostic laboratory which provided extensive metadata for each isolate to facilitate contextual interpretation. Consequently, blinding was not appropriate.

Reporting for specific materials, systems and methods

We require information from authors about some types of materials, experimental systems and methods used in many studies. Here, indicate whether each material, system or method listed is relevant to your study. If you are not sure if a list item applies to your research, read the appropriate section before selecting a response.

Materials & experimental systems

n/a	Involved in the study
☒	☐ Antibodies
☒	☐ Eukaryotic cell lines
☒	☐ Palaeontology and archaeology
☒	☐ Animals and other organisms
☒	☐ Human research participants
☒	☐ Clinical data
☒	☐ Dual use research of concern

Methods

n/a	Involved in the study
☒	☐ ChIP-seq
☒	☐ Flow cytometry
☒	☐ MRI-based neuroimaging
